# Rapidly Progressive Polyneuropathy in a Patient With Monoclonal Gammopathy: A Case Report of POEMS Syndrome and Beyond

**DOI:** 10.1097/MD.0000000000003453

**Published:** 2016-04-22

**Authors:** Chen Wang, Yu-Zhou Guan, Qian-Qian Cai, Wei Su, Dao-Bin Zhou, Jian Li

**Affiliations:** From the Department of Hematology (CW, Q-QC, D-BZ, JL); Department of Neurology (Y-ZG); and Department of Clinical Laboratory (WS), Peking Union Medical College Hospital, Chinese Academy of Medical Sciences and Peking Union Medical College, Beijing, China.

## Abstract

Neuropathy, the dominant clinical feature of POEMS syndrome, is typically distal, symmetric, and slowly progressive with demyelinating changes. After a gradual proximal spread, it usually results in severe muscle weakness and functional disabilities. Cases characterized by acute onset polyneuropathy are rarely described.

In the present report, we describe a 32-year-old male diagnosed as POEMS syndrome, but presenting with a rapidly evolving polyneuropathy. Detailed clinical, electrophysiological, and genetic studies revealed a coexisting underdiagnosed inherited axonal neuropathy, namely Charcot-Marie-Tooth disease 2A2. The patient received lenalidomide-based chemotherapy and consolidated by autologous stem cell transplantation for his POEMS syndrome, which improved the neurological disability.

In most conditions, only 1 cause is responsible for a patient's polyneuropathy. However, an insidious inherited neuropathy can be overlooked, when an acquired condition is present. The case illustrated here, to the best of our knowledge, is the first one with coexistent axonal type Charcot-Marie-Tooth disease and POEMS syndrome, suggesting that an unrecognized inherited neuropathy may change the disease course of a further acquired neuropathy.

## INTRODUCTION

Neuropathy is the most common initial symptom of patients with polyneuropathy, organomegaly, endocrinopathy, monoclonal gammopathy, and skin changes (POEMS) syndrome. Its prominent feature is a slowly progressive disease course with demyelinating electrophysiological changes. Sensory symptoms always precede motor dysfunction, and neuropathic pain has been reported in 10% to 15% cases. Following muscle weakness and atrophy undoubtedly result in functional disabilities that severely impair patients’ quality of life.^1,2^ However, cases with abrupt onset and rapidly evolving polyneuropathy are rarely described. And the underlying reasons remain largely unknown.^3^ Herein, we report a POEMS patient with acute polyneuropathy and explore the causes.

### Case Presentation

A 32-year-old male presented with paresthesia and weakness in his distal extremities. Over time, he developed marked neuropathic pain in his fingers. Simultaneously, muscle weakness spread, and was more predominant in his lower extremities. One month after the onset of these neurological symptoms, he could not dress himself and became wheelchair-bound. No symptoms associated with cranial or autonomic nerves were noted at the time. Serial nerve conduction studies were performed during the disease course, which revealed prolonged distal motor latencies, slowed nerve conduction velocities, and decreased compound muscle action potential amplitudes in a progressive pattern (Table 1).

**TABLE 1:**
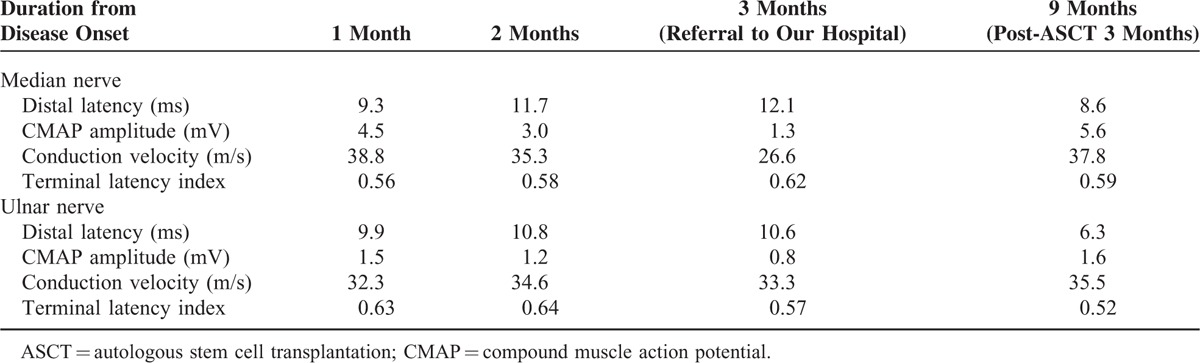
Motor Conduction Studies Before and After Treatment

On referral to our hospital, there were prominent motor (overall neuropathy limitation scale [ONLS]: arm score, 4; leg score, 5) and sensory deficits (neuropathy total symptom score-6 [NTSS-6]: aching pain score, 3.66). Physical examination showed hepatosplenomegaly, generalized lymphadenopathy, sporadic hemangioma across his trunk and skin hyperpigmentation. Laboratory studies revealed: IgG-λ monoclonal protein (detected by serum immunofixation, but not measurable on serum protein electrophoresis), thrombocytosis (371 × 10^9^/L; reference range, 100–300 × 10^9^/L), hypothyroidism (thyrotropin 6.6 μU/mL; reference range, 0.4–4.0 μU/mL), and hyperprolactinemia (22 ng/mL; reference range, 5–20 ng/mL). The cerebrospinal fluid study showed elevated protein (0.9 g/L, reference range, 0–0.4 g/L) and pleocytosis (8 × 10^6^ cells/L, reference range, 0–3 × 10^6^ cells/L). Bone marrow aspirate revealed 0.5% plasma cells with normal morphology (reference range, 0–1.5%). No signs of extravascular volume overload were noted. Osteosclerosis was not seen on x-ray. The clinical constellation suggested a diagnosis of POEMS syndrome. However, the characteristic acute polyneuropathy was atypical, as the median duration from disease onset to inability to walk in POEMS patients was 9 months.^3^ To reach a diagnosis, we measured the serum level of vascular endothelial growth factor (VEGF), which was elevated dramatically (5483 pg/mL; reference range, <600 pg/mL).

**FIGURE 1:**
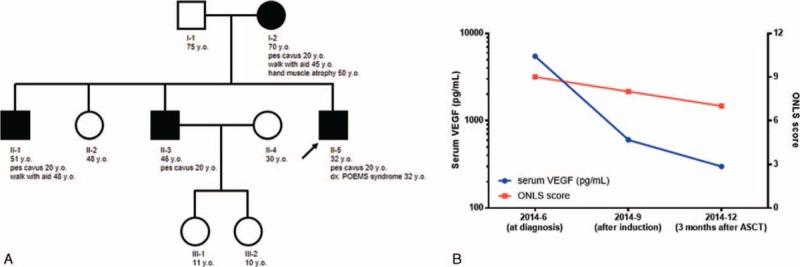
(A) Pedigree of the Charcot-Marie-Tooth disease 2A2 family with MFN2 c.314C>T mutation. The open symbols represent unaffected males (open squares) and unaffected females (open circles), and filled symbols represent affected males (closed squares) and affected females (closed circles), confirmed by genetic tests. The arrow indicates proband. (B) Serial changes of motor dysfunction (assessed by Overall Neuropathy Limitation Scale [ONLS]) and serum vascular endothelial growth factor (VEGF) levels at diagnosis and after treatment (induction therapy, and autologous stem cell transplantation [ASCT]). ASCT = autologous stem cell transplantation; ONLS = Overall Neuropathy Limitation Scale, VEGF = vascular endothelial growth factor.

His past medical history was significant for mild weakness of the distal lower limbs since puberty. Muscle atrophy developed gradually, and pes cavus was noted 20 years prior to our evaluation, although no medical consultation was sought. Further inquiry revealed a similar clinical profile shared by several members of his family (Figure 1), raising the suspicion of an inherited peripheral neuropathy, especially Charcot-Marie-Tooth (CMT) disease.

Overall features of the current disorder were consistent with POEMS syndrome, which requires prompt management. Autologous stem cell transplantation is the preferred treatment for POEMS patients, with universal and durable response.^4^ However, severe neurological dysfunction and poor performance status made the patient too sick to undergo transplantation immediately. Moreover, organomegaly was associated with peripheral blood stem cell mobilization failure.^5^ Therefore, we first gave him 3 courses of lenalidomide (10 mg daily, days 1–21 out of a 28-day cycle) and dexamethasone (40 mg weekly, day 1, 8, 15, 22 out of a 28-day cycle) as an induction therapy, which resulted in an improved neurological function (ONLS: arm score 3; leg score 5) and remission of organomegaly. He then received high-dose melphalan (200 mg/m^2^) with autologous stem cell transplantation as consolidation. Extensive assessments were performed 3 months later. Motor function showed further recovery and the patient could dress part of himself and walk with aid (ONLS: arm score 3; leg score 4), although fine motor skills (e.g., fastening small buttons, zipping up zippers) were still attenuated, which was consistent with the partially improved electrophysiological findings (Table 1). Neuropathic pain was ameliorated (NTSS-6: aching pain score, 1). His IgG-λ monoclonal protein disappeared and serum VEGF level was back to normal (300 pg/mL), as well as other laboratory abnormalities, including thyrotropin, prolactin, and platelet count.

In terms of his suspicious inherited neuropathy, the genetic test covering 27 genes associated with various CMT subtypes, including *AARS*, *DNM2*, *EGR2*, *FGD4*, *FIG4*, *GARS*, *GDAP1*, *GJB1*, *HSPB1*, *HSPB8*, *KIF1B*, *LITAF*, *LMNA*, *MED25*, *MFN2*, *MPZ*, *MTMR2*, *NDRG1*, *NEFL*, *PMP22*, *PRPS1*, *PRX*, *RAB7A*, *SBF2*, *SH3TC2*, *TRPV4* and *YARS*, revealed a heterozygous missense mutation in mitofusin 2 (*MFN2*, c.314C>T), which predicted a substitution of threonine by methionine. This genetic change, which has been described in patients with CMT2A2 with late onset and mild disease, was also detected in other affected members in the pedigree (I-2, II-1, and II-3) and transmitted in an autosomal dominant pattern.^6^ Further screening of monoclonal gammopathy and serum VEGF level did not reveal any abnormality in either neurologically symptomatic or asymptomatic family members. Therefore, the diagnosis of a coexisting CMT2A2 was established. Orthopedic consultation for his pes cavus deformity was obtained and daily stretching exercise was suggested.

## DISCUSSION

To the best of our knowledge, this is the first case with coexistent axonal type CMT and POEMS syndrome, which illustrates several noteworthy issues. In young patients with peripheral neuropathy, even acquired etiologies are revealed, inherited component should also be kept in mind. CMT is a genetically and clinically heterogeneous inherited peripheral neuropathy. Many patients have a long, slowly progressive history, and could be symptomatically mild, and remain undiagnosed until the onset of an acquired disorder. Concerning the peripheral neuropathy in this patient, serial eletrophysiological studies gave us a unique opportunity to depict its progression in the presence of both inherited and acquired disorders. Simultaneously progressed demyelination and axonal degradation indicate a mixed neuropathy rather than ones that developed sequentially. Microvascular hyperpermeability induced by VEGF, with blood–nerve barrier opening, is thought to underlie the demyelinating polyneuropathy of POEMS syndrome. And, the chronic injury of nerve vasculature is consistent with a prolonged clinical course.^7,8^ In the present case, a pre-existing axonal neuropathy (CMT2A2), although relatively silent that did not interfere the daily life of this patient, rendered the nerve more susceptible to further injury. This might be responsible for an atypical peripheral neuropathy in POEMS, that is, acute worsening and rapid exacerbation. In terms of treatment, initial disease control with lenalidomide, followed by autologous stem cell transplantation is safe and effective for POEMS syndrome.^9^ Although no disease-modifying therapy is available for CMT, orthopedic foot surgery and daily stretching exercise are helpful and could improve the quality of life, which is only made possible by providing a correct diagnosis. Moreover, accumulating evidence indicate that a cryptic and superimposed inflammatory process exists in a subgroup of patients with CMT disease, which may explain the considerable phenotypic heterogeneity. Clinical benefits were observed in sporadic cases treated with anti-inflammatory agents.^10^ Therefore, it will be of great interest to see whether the peripheral neuropathy of this patient could be further improved with our treatment in a long-term view.
